# TRI-SCORE based risk stratification across contemporary treatment strategies for isolated tricuspid regurgitation: a multicenter retrospective analysis

**DOI:** 10.3389/fcvm.2026.1892142

**Published:** 2026-08-26

**Authors:** Sara Gharehdaghi, Liesbeth Rosseel, Monika Beles, Marc Vanderheyden, Bernard Stockman, Filip Casselman, Raffaella Mistrulli, Ivan Degrieck, Karel Van Praet, Jerrold Spapen, Sofie Brouwers, Frank Van Praet, Martin Penicka, Guy Van Camp

**Affiliations:** 1 Cardiovascular Center, AZORG Hospital, Aalst, Belgium; 2 Doctoral School, Semmelweis University, Budapest, Hungary; 3 Department of Clinical and Molecular Medicine, Sapienza University of Rome, Roma, Italy

**Keywords:** right heart failure, risk stratification, transcatheter tricuspid edge-to-edge repair (T-TEER), tricuspid regurgitation (TR), TRI-SCORE, valve surgery

## Abstract

**Background:**

The TRI-SCORE is a disease-specific risk model developed to predict outcomes after isolated tricuspid valve surgery. Although its prognostic value has been demonstrated across contemporary treatment strategies, independent external validation in multicenter cohorts across a broader clinical and etiological spectrum remains limited. We evaluated the prognostic performance of the TRI-SCORE in patients with isolated ≥ moderate tricuspid regurgitation (TR) treated with medical therapy, surgery, or transcatheter tricuspid edge-to-edge repair (T-TEER).

**Methods:**

In this retrospective multicenter study, 104 patients with isolated ≥ moderate TR treated between 2010 and 2024 were included. Patients underwent medical therapy (*n* = 31), isolated tricuspid valve surgery (*n* = 40), or T-TEER (*n* = 33). The primary endpoint was all-cause mortality, and the secondary endpoint was a composite of all-cause mortality or heart failure hospitalization. Multivariable Cox proportional hazards models included TRI-SCORE and selected clinically relevant covariates.

**Results:**

The median age was 77.0 years (IQR, 69.8–83.0), 62% of patients were women, and the median follow-up was 45 months (IQR, 27–63). During follow-up, all-cause mortality occurred in 37 (36%), and the composite endpoint in 40 (38%). One-year all-cause mortality increased across TRI-SCORE categories (7%, 13%, and 33% in the low-, intermediate-, and high-risk groups, respectively; *P* = 0.03). Baseline TRI-SCORE independently predicted all-cause mortality (HR 1.23; 95% CI 1.08–1.40) and the composite endpoint (HR 1.34; 95% CI 1.17–1.52). At 1 year, TR was reduced to < moderate severity in 60% of medically treated patients, 91% of surgically treated patients, and 68% of T-TEER-treated patients. Although surgery was associated with a lower risk of the composite endpoint (HR 0.39; 95% CI 0.17–0.89), Kaplan–Meier analysis showed no significant survival difference among treatment groups.

**Conclusions:**

In this multicenter real-world cohort of patients with isolated ≥ moderate TR, the TRI-SCORE provided independent prognostic information across medical therapy, surgery, and T-TEER. These findings provide external validation of the TRI-SCORE in a multicenter cohort representing a broader clinical and etiological spectrum than previously evaluated, supporting its use for baseline risk stratification across contemporary management strategies. Treatment comparisons should be interpreted cautiously because of the observational study design.

## Graphical Abstract

**Figure:**
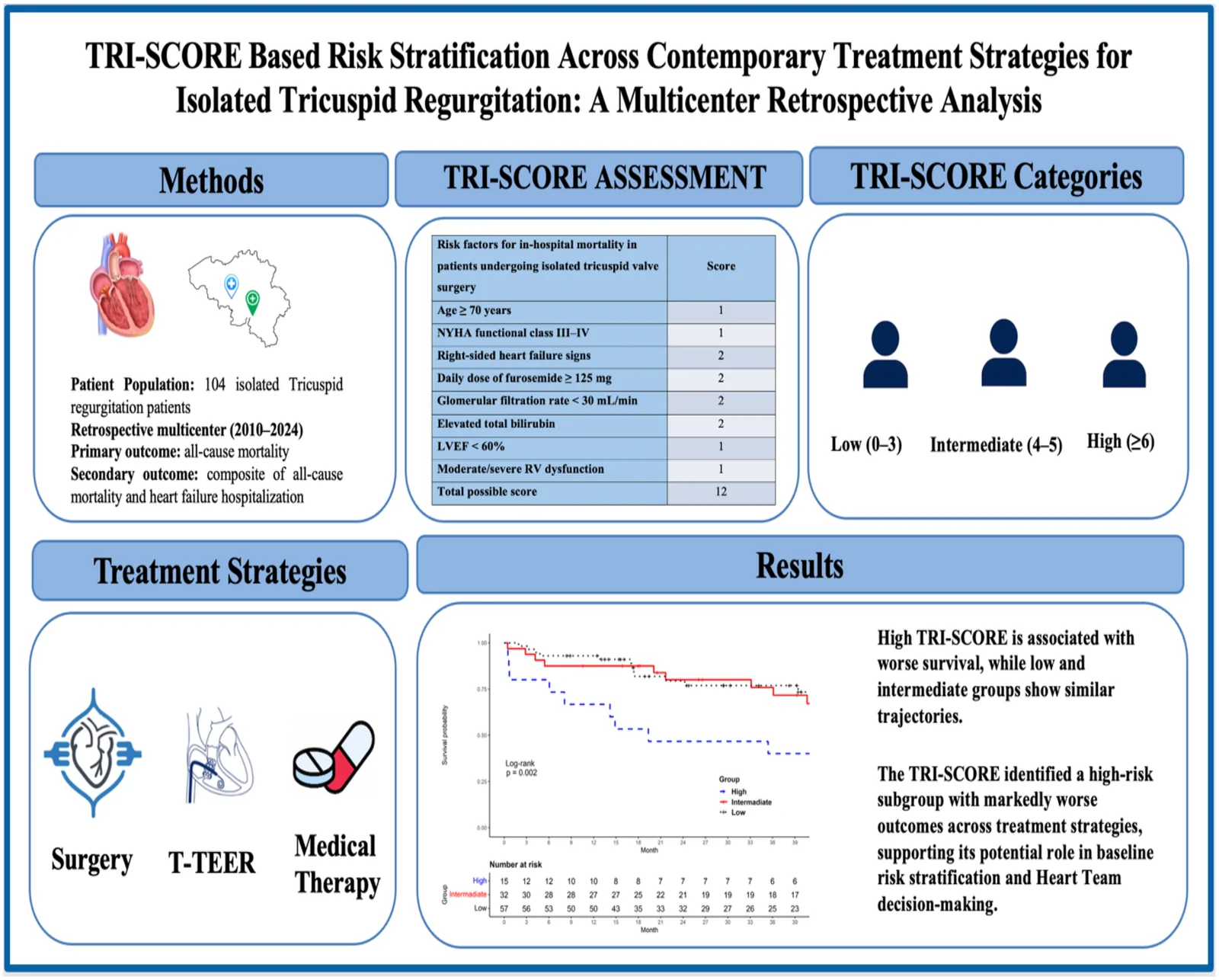

## Introduction

Tricuspid regurgitation (TR) is increasingly recognized as a prevalent valvular heart disease with important prognostic implications. Previously considered a benign sequela of left-sided heart disease, isolated functional TR has been shown to be associated with increased morbidity and mortality (1–4).

Despite its clinical impact, TR has historically been underdiagnosed and undertreated (5, 6), largely because of delayed referral and the perceived high operative risk associated with isolated tricuspid valve surgery. In recent years, advances in perioperative management, minimally invasive surgical techniques, and the development of transcatheter tricuspid edge-to-edge repair (T-TEER) have substantially expanded therapeutic options (7, 8). Among these, T-TEER has demonstrated favorable procedural safety and efficacy in patients deemed inoperable or at high surgical risk (9–13). Although randomized trials such as TRILUMINATE have demonstrated significant improvements in symptoms, functional status, and quality of life following T-TEER, a reduction in mortality has not yet been demonstrated (10). These findings emphasize that improved patient selection and optimal timing of intervention remain essential, underscoring the need for robust disease-specific risk stratification tools.

In this rapidly evolving therapeutic landscape, accurate risk stratification is essential to support multidisciplinary Heart Team decision-making regarding both the timing and selection of intervention. The TRI-SCORE is a composite risk model that integrates eight clinical, biochemical, and echocardiographic parameters and was originally developed and validated to predict in-hospital mortality after isolated tricuspid valve surgery (14). Subsequent studies confirmed its prognostic value in surgical populations (15) and further demonstrated its applicability in patients undergoing transcatheter tricuspid interventions, including T-TEER (16–18). More recently, investigators from the international TRIGISTRY registry showed that increasing TRI-SCORE values were associated with progressively worse outcomes among patients managed conservatively, surgically, or with transcatheter intervention, and that the benefit of intervention appeared greatest in patients with lower baseline risk (19, 20).

Despite these advances, several important knowledge gaps remain. Previous studies have established the prognostic value of the TRI-SCORE in patients undergoing isolated tricuspid valve surgery, transcatheter tricuspid interventions, and, more recently, in the international TRIGISTRY registry encompassing conservative, surgical, and transcatheter treatment strategies (14–20). However, independent external validation in contemporary multicenter cohorts representing a broader clinical and etiological spectrum than previously evaluated remains limited, particularly among patients with isolated ≥ moderate tricuspid regurgitation and diverse disease etiologies encountered in routine clinical practice (14–20). Furthermore, longitudinal changes in residual tricuspid regurgitation following different management strategies have not been well characterized in contemporary multicenter real-world cohorts.

Accordingly, this multicenter study aimed to provide an independent external validation of the TRI-SCORE in a contemporary multicenter real-world cohort of patients with isolated ≥ moderate tricuspid regurgitation managed with medical therapy, isolated tricuspid valve surgery, or T-TEER. Specifically, we sought to evaluate the prognostic performance of the TRI-SCORE for all-cause mortality and the composite endpoint of all-cause mortality or heart failure hospitalization. Secondary objectives were to characterize longitudinal changes in residual tricuspid regurgitation following each treatment strategy and to descriptively assess clinical outcomes across treatment groups.

## Methods

### Study design and population

We performed a retrospective, observational, multicenter cohort study at Onze-Lieve-Vrouwziekenhuis (OLV) and Algemeen Stedelijk Ziekenhuis (ASZ) Hospitals (Aalst, Belgium). Consecutive adult patients with isolated ≥ moderate tricuspid regurgitation (TR) who were managed medically, surgically, or with T-TEER between January 2010 and April 2024 were identified from the institutional echocardiography and clinical databases of the participating centers. Both outpatient and hospitalized patients were eligible for inclusion. Patients were included only if complete baseline data required for TRI-SCORE calculation were available. For hospitalized patients, the baseline echocardiographic assessment corresponded to the clinical evaluation performed after clinical stabilization, at which the Heart Team or treating physicians established the treatment strategy and the baseline TRI-SCORE was assessed.

### Ethical approval

The study was approved by the institutional ethics committee of OLV and ASZ Hospitals (approval number: 2025/006). Owing to the retrospective and non-interventional design of the study, the requirement for written informed consent was waived. The study was conducted in accordance with the principles of the Declaration of Helsinki.

### Treatment strategy selection

Treatment strategy (medical therapy, surgery, or T-TEER) was determined by the local multidisciplinary Heart Team and treating physicians based on a comprehensive clinical evaluation. Decision-making incorporated multiple factors, including surgical risk (e.g., EuroSCORE II), anatomical suitability for intervention, the severity of tricuspid regurgitation, right ventricular function, comorbid conditions, frailty, and patient preference. Procedural feasibility and evolving institutional expertise, particularly for transcatheter therapies, were also considered.

Baseline was defined as the clinical time point at which the treatment strategy was established and the TRI-SCORE was assessed. For patients undergoing isolated tricuspid valve surgery or T-TEER, baseline corresponded to the date of intervention, using the most recent preprocedural clinical, laboratory, and echocardiographic data available for TRI-SCORE calculation. For medically managed patients, baseline was defined as the date of the clinical evaluation at which isolated ≥ moderate tricuspid regurgitation was documented and conservative management was selected. Follow-up was calculated from this baseline date until death, the last clinical contact, or the end of the study period.

The primary objective of the study was to independently validate the prognostic performance of the baseline TRI-SCORE in a contemporary multicenter real-world cohort of patients with isolated ≥ moderate tricuspid regurgitation managed with medical therapy, isolated tricuspid valve surgery, or T-TEER, including a broader clinical and etiological spectrum than previously evaluated. Secondary objectives were to characterize longitudinal changes in residual tricuspid regurgitation following each treatment strategy and to descriptively assess clinical outcomes across treatment groups. The study was not designed to estimate the causal effects of treatment strategies. Accordingly, treatment strategy was analyzed according to the management approach selected in routine clinical practice. Because treatment allocation was not randomized but instead reflected individualized clinical judgment, comparisons between treatment groups are subject to confounding by indication and selection bias and should be interpreted as exploratory and hypothesis-generating rather than causal estimates of treatment effects.

### Exclusion criteria

Exclusion criteria included severe pulmonary hypertension, defined as systolic pulmonary artery pressure (sPAP) > 70 mmHg; left ventricular ejection fraction (LVEF) < 40%; and active infective endocarditis. In addition, patients with significant left-sided valvular disease were excluded, defined as moderate or greater mitral or aortic stenosis or regurgitation, or the presence of untreated, clinically relevant left-sided valvular disease requiring intervention. Patients with heart failure with preserved ejection fraction (HFpEF) were not excluded, provided that the LVEF was ≥40% and TR was considered the predominant valvular pathology. Chronic lung disease was not an explicit exclusion criterion. However, patients with advanced pulmonary disease contributing to severe pulmonary hypertension (sPAP >70 mmHg) were excluded, as described above.

### Echocardiographic assessment of tricuspid regurgitation

TR severity was assessed using transthoracic echocardiography performed as part of routine clinical care at each participating center. Grading of TR severity was based on contemporaneous guideline recommendations and incorporated an integrated, multiparametric approach that included both qualitative and semiquantitative measures. Parameters considered included vena contracta width, jet area, hepatic vein systolic flow reversal, tricuspid annular dilation, and right atrial and right ventricular enlargement, together with the overall visual assessment by experienced echocardiographers. For consistency across the retrospective study period, TR severity was classified using the historical four-grade classification (Grades 1–4), in which Grade 1 represented mild TR, Grades 2 and 3 represented moderate TR, and Grade 4 represented severe TR. Given the retrospective nature of the study and the inclusion period beginning in 2010, the contemporary expanded five-grade classification of TR severity (Grade 1, mild; Grade 2, moderate; Grade 3, severe; Grade 4, massive; and Grade 5, torrential TR) was not systematically applied across the cohort. Consequently, all analyses were performed using the historical four-grade classification to ensure consistency across the entire study population, in accordance with the current ESC/EACTS Valvular Heart Disease Guidelines (8).

All echocardiographic assessments were performed locally without a centralized core laboratory, and measurements were based on standard clinical reports at each institution. Assessment of residual TR severity at 1-year follow-up was performed only in patients with available follow-up echocardiographic examinations and therefore reflects echocardiographic outcomes rather than the entire study cohort. Accordingly, analyses of longitudinal changes in TR severity were restricted to patients with available follow-up echocardiographic examinations and should be interpreted independently of the survival analyses, which included the entire study cohort and were based on time-to-event methodology. In addition to grading TR severity, TR etiology was classified as functional, primary (organic, including cardiac implantable electronic device [CIED]-related), or mixed according to contemporary guideline definitions. Functional TR was further subclassified as ventricular or atrial TR. Etiological classification was based on a comprehensive review of baseline clinical records, echocardiographic findings, device history, and operative reports, when applicable.

### TRI-SCORE assessment

The TRI-SCORE was calculated for each patient using the original scoring system described by Dreyfus et al. (14), with variables derived from baseline clinical, laboratory, and echocardiographic data. Patients were stratified according to their baseline TRI-SCORE into low- (0–3), intermediate- (4–5), and high-risk (≥6) categories. Assessment of right ventricular (RV) dysfunction was based on comprehensive transthoracic echocardiographic evaluation performed in routine clinical practice according to contemporary guideline recommendations. RV systolic function was assessed using an integrated multiparametric approach incorporating tricuspid annular plane systolic excursion (TAPSE), RV fractional area change (FAC), tissue Doppler-derived tricuspid annular S' velocity, and qualitative visual assessment by experienced echocardiographers, in accordance with contemporary recommendations for right heart echocardiographic assessment (21). For TRI-SCORE calculation, RV dysfunction was classified according to the presence of moderate or severe RV dysfunction based on the overall echocardiographic assessment. Elevated total bilirubin was defined as a value exceeding the institutional laboratory upper reference limit. All TRI-SCORE components and their corresponding point allocations were applied in accordance with the original model (14) using the official online TRI-SCORE calculator (https://www.tri-score.com).

### Outcomes and statistical analysis

The primary outcome was all-cause mortality during follow-up. The secondary outcome was a composite of all-cause mortality and heart failure–related hospitalization, defined as admission for worsening heart failure (New York Heart Association [NYHA] class ≥ III) requiring escalation of loop diuretic therapy. For each time-to-event analysis, patients who did not experience the corresponding endpoint were censored at the date of their last clinical follow-up or at the end of the study period, whichever occurred first. Given the retrospective study design, no independent event adjudication committee was available.

Normality of continuous variables was assessed graphically using quantile–quantile (Q–Q) plots. Because several variables deviated from a normal distribution and the sample sizes of the individual treatment groups were relatively small, nonparametric statistical methods were used throughout the analyses. Continuous variables are presented as medians with interquartile ranges (IQRs), and categorical variables as counts and percentages. Continuous variables were compared using the Kruskal–Wallis test or the Wilcoxon rank-sum test, as appropriate. Longitudinal within-patient comparisons of tricuspid regurgitation severity between baseline, 30 days, and 1 year were performed using the paired Wilcoxon signed-rank test. Categorical variables were compared using the *χ*^2^ test with Yates' continuity correction or Fisher's exact test, as appropriate. For contingency tables larger than 2 × 2 with sparse expected cell counts, the Fisher–Freeman–Halton exact test was used.

Kaplan–Meier curves were generated to estimate survival according to TRI-SCORE category and treatment modality and were compared using the log-rank test. Cox proportional hazards regression analysis was used to evaluate the associations of TRI-SCORE and clinically relevant covariates with the study outcomes. Given the limited sample size and number of outcome events, the multivariable models were intentionally restricted to a limited number of covariates to reduce the risk of overfitting and unstable coefficient estimates.

First, univariable Cox proportional hazards regression analyses were performed for clinically relevant candidate variables. Variables associated with the outcome at *P* < 0.05 were considered eligible for inclusion in the multivariable model. Among the eligible candidate variables, age, sex, and treatment strategy were selected for inclusion in the initial multivariable model based on their statistical association with the outcome and their clinical relevance.

Although EuroSCORE II was significantly associated with the outcome in the univariable analysis, it was deliberately excluded from the multivariable model because of its conceptual overlap with TRI-SCORE, as both are composite risk scores that capture similar prognostic information. Including both scores in the same model could have introduced multicollinearity and made interpretation of their individual effects more difficult.

The initial multivariable model included TRI-SCORE together with the selected covariates (age, sex, and treatment strategy). Because evaluation of the independent prognostic value of TRI-SCORE was the primary objective of the study, TRI-SCORE was retained in all multivariable models irrespective of statistical significance. The remaining eligible covariates were sequentially removed using manual backward elimination (*P* > 0.05) to derive the final parsimonious model.

The proportional hazards assumption was assessed using Schoenfeld residuals. To illustrate the results of the final Cox regression models, model-based predicted survival curves were generated.

Although TRI-SCORE was modeled as a continuous variable in the Cox regression analyses, the predefined risk categories (low, intermediate, and high) proposed by Dreyfus et al. were used only for Kaplan–Meier analyses and the graphical presentation of model-based predicted survival curves to facilitate clinical interpretation. Statistical significance was defined as a two-sided *P* < 0.05. All analyses were performed using R version 4.1.0 (R Foundation for Statistical Computing, Vienna, Austria). No imputation of missing data was performed because there were no missing values for the variables included in the multivariable analyses. One-year mortality analyses included patients with at least 365 days of potential follow-up or those who died within the first year after baseline.

## Results

### Baseline characteristics and treatment strategies

A total of 104 patients with isolated ≥ moderate TR were included. Of these, 40 patients (38%) underwent surgical intervention (repair in 17 and replacement in 23), 33 (32%) underwent T-TEER, and 31 (30%) received medical therapy. In the surgical group, 36 patients (90%) underwent a minimally invasive procedure, whereas 4 (10%) underwent surgery via sternotomy. The median follow-up was 45 months (IQR, 27–63 months).

Among the 104 patients, functional TR was the predominant etiology (76.0%), followed by mixed TR (12.5%) and primary and CIED-related (organic) TR (11.5%) (Table 1). Among patients with functional TR, ventricular functional TR was the most common subtype (59/79, 74.7%), followed by atrial functional TR (20/79, 25.3%).

**Table 1:** Baseline clinical characteristics of patients with isolated tricuspid regurgitation according to treatment strategy.

Criterion	Total (*N*=104)	Medical (*N*=31)	Surgery (*N*=40)	T-TEER (*N*=33)	*P*-value
Age*, years (median [IQR])	77.0 [69.8–83.0]	79.0 [74.5–87.5]	69.0 [64–77.5]	80.0 [76–84]	<0.01
Male sex, *n* (%)	40 (38.5%)	13 (41.9%)	11 (27.5%)	16 (48.5%)	0.17
Length, cm	166 [161–170]	167 [162–172]	167 [160–170]	165 [162–169]	0.8
Weight, kg	72.0 [62.8–83.4]	74.0 [66–89]	72.0 [57.8–78]	72.0 [65–84.6]	0.18
Arterial Hypertension, *n* (%)	48 (46.2%)	21 (67.7%)	18 (45.0%)	9 (27.3%)	< 0.01
Diabetes mellitus, *n* (%)	18 (17.3%)	5 (16.1%)	10 (25.0%)	3 (9.1%)	0.2
Atrial fibrillation, *n* (%)	83 (79.8%)	23 (74.2%)	28 (70.0%)	32 (97.0%)	0.01
Chronic kidney disease, *n* (%)	29 (27.9%)	8 (25.8%)	10 (25.0%)	11 (33.3%)	0.7
eGFR*, mL/min/1.73m²	48.5 [37–63]	47 [36–60]	51 [38–87.5]	48 [37–57]	0.262
Coronary artery disease, *n* (%)	29 (27.9%)	10 (32.3%)	11 (27.5%)	8 (24.2%)	0.77
Previous CABG, *n* (%)	16 (15.4%)	7 (22.6%)	6 (15.0%)	3 (9.1%)	0.32
Previous MV surgery, *n* (%)	31 (29.8%)	14 (45.2%)	13 (32.5%)	4 (12.1%)	0.01
ACE-inhibitor use, *n* (%)	29 (27.9%)	10 (32.3%)	10 (25.0%)	9 (27.3%)	0.8
ARB use, *n* (%)	16 (15.4%)	5 (16.1%)	5 (12.5%)	6 (18.2%)	0.8
Beta-blocker use, *n* (%)	77 (74.0%)	23 (74.2%)	23 (57.5%)	31 (93.9%)	< 0.01
MRA use, *n* (%)	67 (64.4%)	19 (61.3%)	29 (72.5%)	19 (57.6%)	0.38
Diuretic use, *n* (%)	75 (72.1%)	22 (71.0%)	27 (67.5%)	26 (78.8%)	0.55
TRI-SCORE (baseline)	3.0 [1–4.25]	3.0 [1–4]	4.0 [2–5]	2.0 [1–4]	0.04
Low TRI-SCORE, *n* (%)	57 (54.8%)	19 (61.3%)	15 (37.5%)	23 (69.7%)	0.07
Intermediate TRI-SCORE, *n* (%)	32 (30.8%)	9 (29.0%)	17 (42.5%)	6 (18.2%)	0.07
High TRI-SCORE, *n* (%)	15 (14.4%)	3 (9.7%)	8 (20.0%)	4 (12.1%)	0.07
EuroSCORE II**	3.23 [1.69–5.95]	3.17 [1.99–4.03]	6.15 [1.75–12.6]	2.36 [1.59–3.38]	< 0.01
TR etiology					0.005
Functional TR, *n* (%)	79 (76.0%)	27 (87.1%)	26 (65.0%)	26 (78.8%)	
Ventricular***	59 (56.7%)	17 (54.8%)	21 (52.5%)	21 (63.6%)	
Atrial***	20 (19.2%)	10 (32.3%)	5 (12.5%)	5 (15.2%)	
Primary and CIED-related (organic)	12 (11.5%)	2 (6.5%)	9 (22.5%)	1 (3.0%)	
Mixed	13 (12.5%)	2 (6.5%)	5 (12.5%)	6 (18.2%)	
Severe TR (grade 4), *n* (%)	58 (55.8%)	5 (16.1%)	27 (67.5%)	26 (78.8%)	<0.01

Values are presented as median (interquartile range [IQR]) or number (%), as appropriate. Variables marked with an asterisk (*) are components of the TRI-SCORE. TR was classified as functional, primary and CIED-related (organic), or mixed. Functional TR was further subclassified as ventricular or atrial TR. The overall distribution of TR etiology across treatment groups was compared using the Fisher–Freeman–Halton exact test (*P* = 0.005). Owing to the small numbers of patients in the individual etiologic subgroups, statistical comparisons were performed only across the three main TR etiology categories.

** EuroSCORE II incorporates age, sex, renal function, extracardiac arteriopathy, poor mobility, previous cardiac surgery, chronic lung disease, active endocarditis, critical preoperative status, New York Heart Association functional class, Canadian Cardiovascular Society angina class, left ventricular ejection fraction, recent myocardial infarction, pulmonary hypertension, urgency of surgery, and procedure-related factors to estimate operative mortality risk.

*** Percentages for ventricular and atrial TR were calculated using the total number of patients in each treatment group as the denominator: overall cohort, *N* = 104; medical therapy, *N* = 31; surgery, *N* = 40; and T-TEER, *N* = 33.

ACE inhibitor, angiotensin-converting enzyme inhibitor; ARB, angiotensin II receptor blocker; CABG, coronary artery bypass grafting; CIED, cardiac implantable electronic device; eGFR, estimated glomerular filtration rate; EuroSCORE II, European System for Cardiac Operative Risk Evaluation II; MRA, mineralocorticoid receptor antagonist; MV, mitral valve; T-TEER, transcatheter tricuspid edge-to-edge repair; TR, tricuspid regurgitation; TRI-SCORE, Tricuspid Regurgitation Risk Score.

Baseline characteristics are summarized in Table 1. The median age was 77 years (IQR, 69.8–83.0), and 62% of patients were women. Atrial fibrillation was highly prevalent (80%). Chronic kidney disease was common, with a median estimated glomerular filtration rate (eGFR) of 48.5 mL/min/1.73 m^2^ (IQR, 37.0–63.0). Primary and CIED-related (organic) TR was more prevalent in the surgical group (22.5%) than in the T-TEER and medical therapy groups.

Patients treated surgically were significantly younger than those undergoing T-TEER or receiving medical therapy (median age, 69 vs. 80 and 79 years, respectively; *p* < 0.01) and more frequently had preserved renal function (median eGFR, 51 mL/min/1.73 m^2^ [IQR, 37.8–87.5]). Despite their younger age, surgical patients had the highest baseline TRI-SCORE, indicating greater predicted operative risk according to the TRI-SCORE model. Baseline TR severity was also greatest in the surgical and T-TEER groups, whereas the highest baseline TRI-SCORE was observed in the surgical group.

### TRI-SCORE distribution and baseline risk profile

At baseline, 57 patients (55%) were classified as low risk (TRI-SCORE 0–3), 32 (31%) as intermediate risk (4–5), and 15 (14%) as high risk (≥6). The distribution of individual TRI-SCORE components across treatment groups is presented in Supplementary Table S2. Baseline characteristics stratified by TRI-SCORE category are summarized in Supplementary Table S3.

As expected, several variables that constitute components of the TRI-SCORE, including age and estimated glomerular filtration rate (eGFR), differed significantly across the predefined risk categories. These differences reflect the construction of the score and should not be interpreted as independent associations. Accordingly, the interpretation focused primarily on baseline variables not incorporated into the TRI-SCORE.

Among the baseline variables not included in the TRI-SCORE, EuroSCORE II differed significantly across the TRI-SCORE categories, with progressively higher values observed in the higher-risk groups, reflecting greater estimated operative risk.

## Clinical outcomes

During follow-up, all-cause mortality occurred in 37 patients (36%): 39% in the medical therapy group, 38% in the surgical group, and 30% in the T-TEER group (overall group comparison, *P* = 0.74). The composite endpoint of all-cause mortality or heart failure hospitalization occurred in 40 patients (38%), with event rates of 45%, 38%, and 33% in the medical therapy, surgical, and T-TEER groups, respectively (overall group comparison, *P* = 0.62).

All-cause mortality during follow-up increased markedly across the TRI-SCORE categories, occurring in 25% of low-risk, 34% of intermediate-risk, and 80% of high-risk patients (*p* < 0.01). At 1 year, the composite endpoint of all-cause mortality or heart failure hospitalization occurred in 11%, 16%, and 47% of low-, intermediate-, and high-risk patients, respectively (*p* < 0.01). Similarly, one-year all-cause mortality increased across the TRI-SCORE categories (7%, 13%, and 33%, respectively; *P* = 0.03).

**Figure 1:**
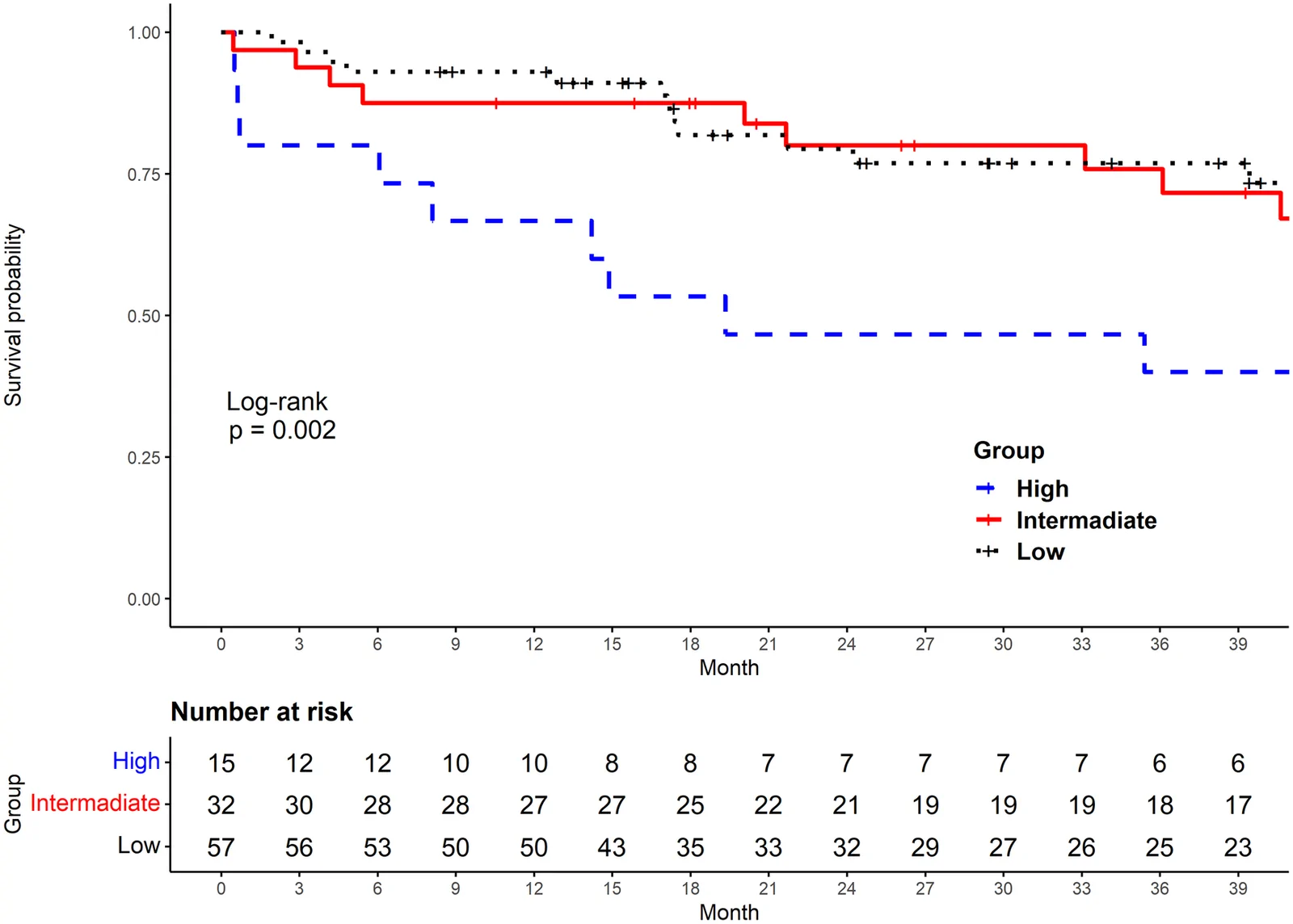
Kaplan–Meier curves for all-cause mortality stratified by TRI-SCORE category. Kaplan–Meier survival curves for all-cause mortality according to the baseline TRI-SCORE categories: low (0–3), intermediate (4–5), and high (≥6). A significant difference in survival was observed across the groups (log-rank *P* = 0.002). The high-risk TRI-SCORE category demonstrated markedly worse survival than the low- and intermediate-risk categories. In contrast, the survival trajectories of the low- and intermediate-risk groups were more closely aligned, with limited separation during follow-up. Numbers at risk at each time point are displayed below the *x*-axis.

**Figure 2:**
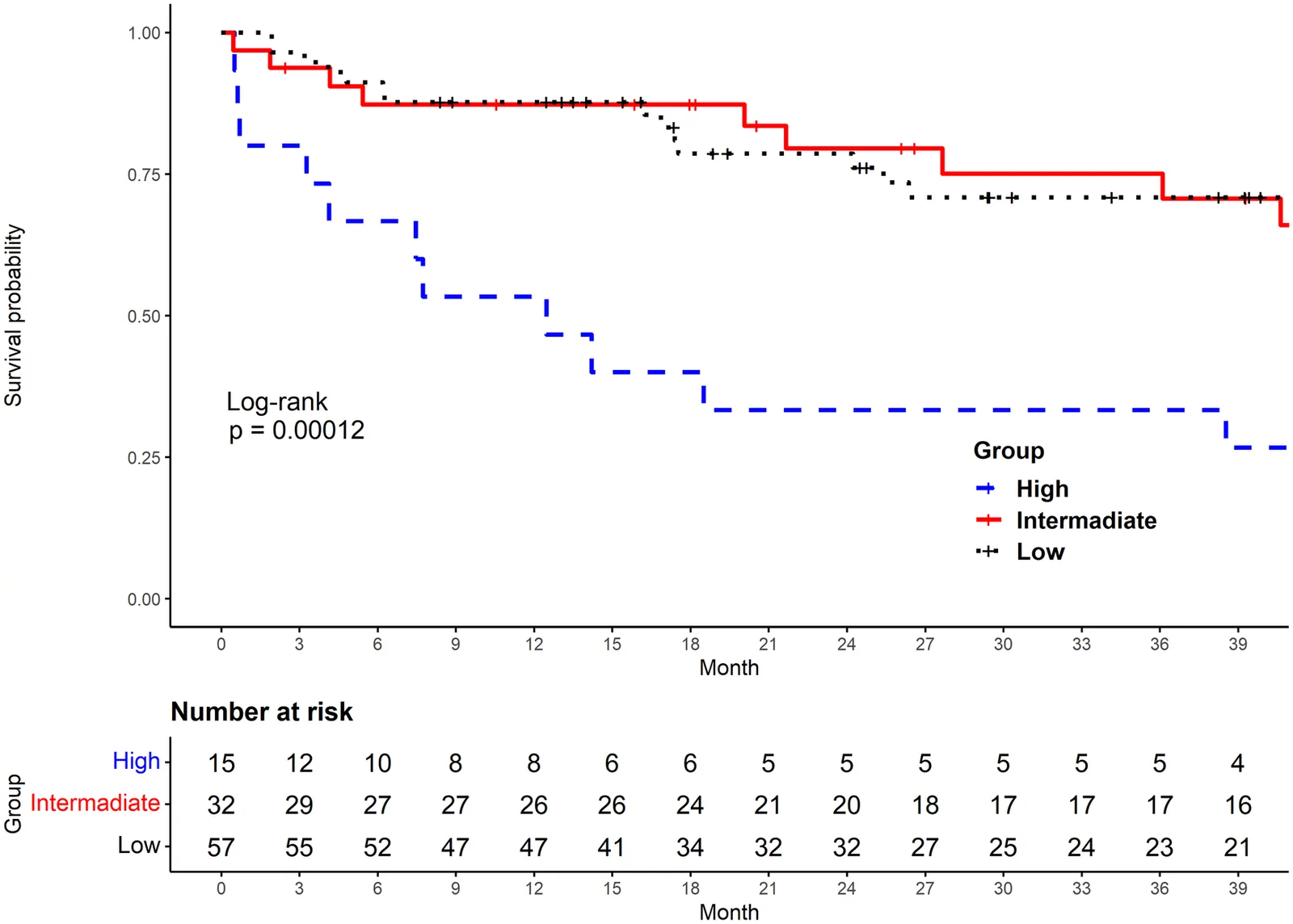
Kaplan–Meier curves for the composite endpoint (all-cause mortality or heart failure hospitalization) stratified by TRI-SCORE category. Kaplan–Meier curves for the composite endpoint of all-cause mortality or heart failure hospitalization according to the baseline TRI-SCORE categories: low (0–3), intermediate (4–5), and high (≥6). A significant difference in event rates was observed across the groups (log-rank *P* = 0.00012). Patients in the high-risk TRI-SCORE category exhibited substantially higher event rates during follow-up. The low- and intermediate-risk groups demonstrated more similar event trajectories, with less pronounced separation than the high-risk group. Numbers at risk at each time point are shown below the *x*-axis.

**Figure 3:**
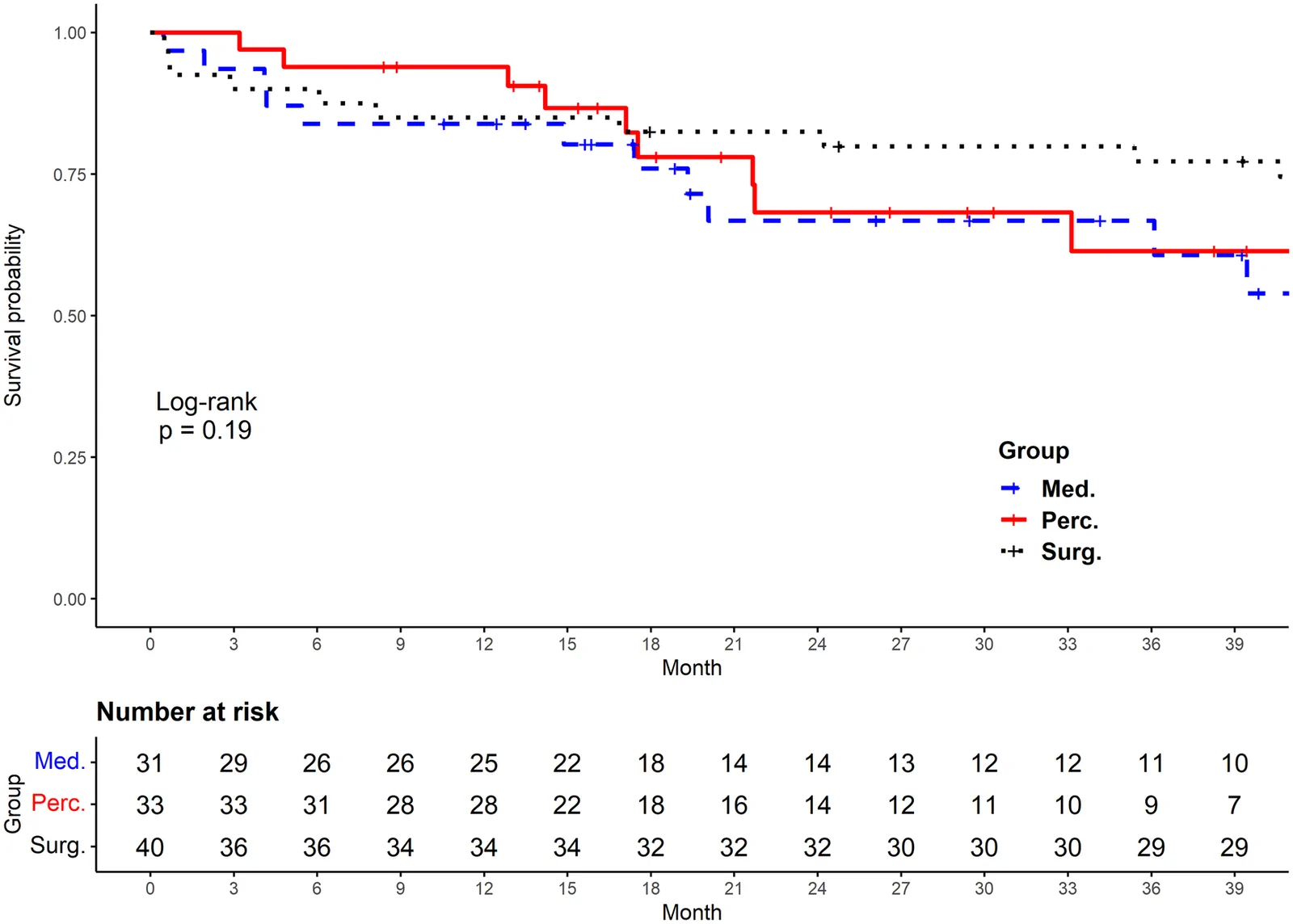
Kaplan–Meier survival curves for all-cause mortality stratified by treatment strategy. Survival estimates are shown for patients receiving medical therapy (blue), transcatheter tricuspid edge-to-edge repair (T-TEER) (red), and surgical intervention (black). Survival curves were compared using the log-rank test and showed no statistically significant difference in all-cause mortality among the treatment groups (log-rank *P* = 0.19). Numbers at risk at each time point are displayed below the *x*-axis.

**Figure 4:**
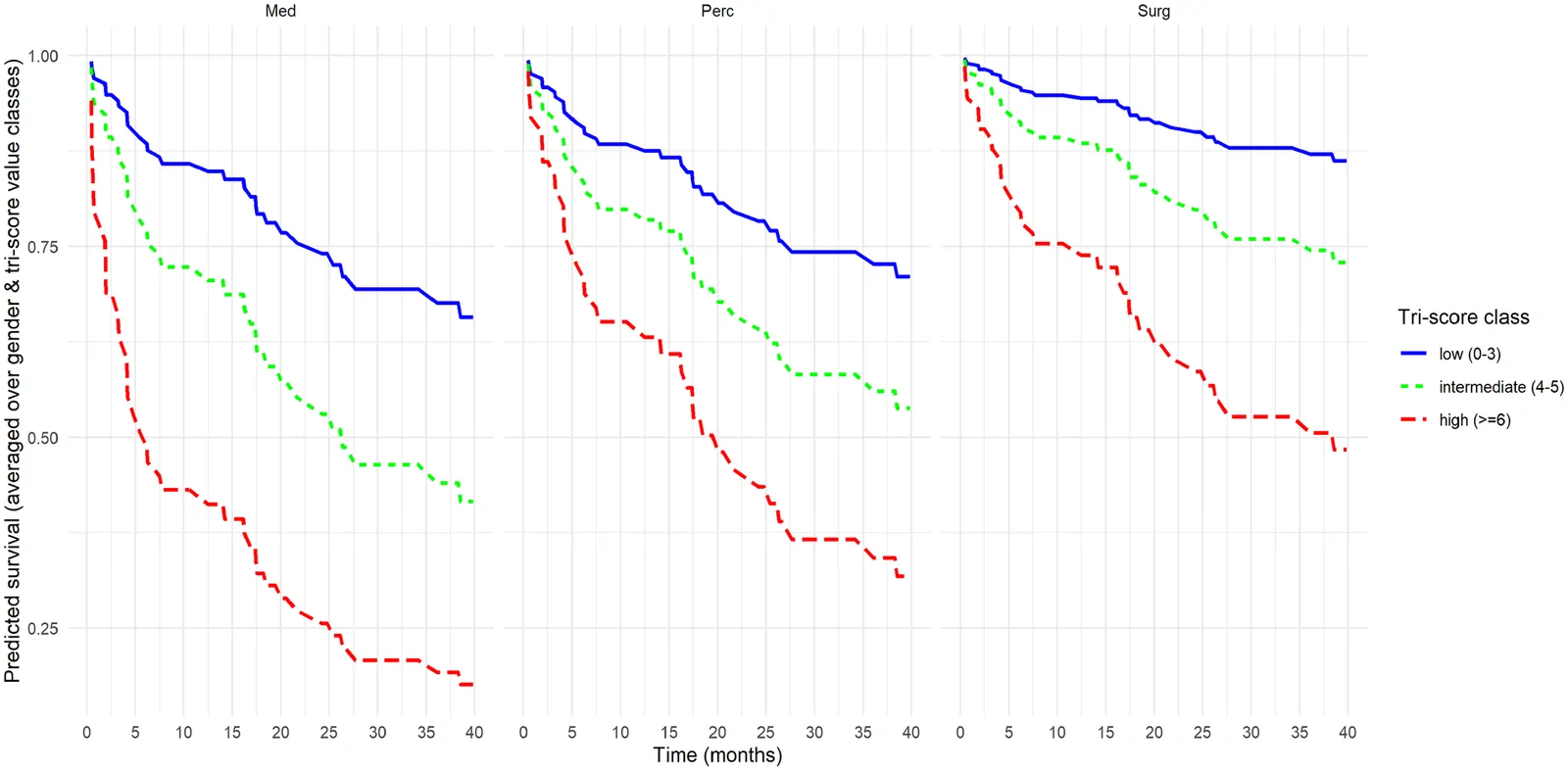
Model-based predicted survival for the composite endpoint (all-cause mortality or heart failure hospitalization) according to treatment strategy and TRI-SCORE category. Predicted survival curves were derived from the final multivariable Cox proportional hazards model, which included TRI-SCORE category, treatment strategy, and sex. These curves represent model-based survival estimates conditional on the included covariates and should not be interpreted as directly observed Kaplan–Meier estimates. Across TRI-SCORE categories, the model-based estimates suggested more favorable predicted survival among surgically treated patients, followed by patients undergoing transcatheter tricuspid edge-to-edge repair (T-TEER) and those receiving medical therapy. Because treatment allocation was nonrandomized and influenced by baseline clinical characteristics, these comparisons should be interpreted as exploratory rather than causal.

Kaplan–Meier survival analyses demonstrated significant separation among the TRI-SCORE categories for both the primary and composite endpoints (Figures 1, 2). In the final parsimonious multivariable Cox proportional hazards model, baseline TRI-SCORE, increasing age, and female sex were independently associated with all-cause mortality (Supplementary Table S4). In exploratory multivariable analyses, surgical treatment was associated with a lower risk of the composite endpoint (HR, 0.39; 95% CI, 0.17–0.89). However, given the observational study design, baseline imbalances, and limited sample size, this finding should be interpreted cautiously and should not be considered evidence of treatment superiority (Supplementary Table S5). Kaplan–Meier survival analyses stratified by treatment strategy showed no significant differences in outcomes among the treatment groups (Figure 3). Model-based predicted survival curves derived from the Cox proportional hazards model incorporating TRI-SCORE, treatment strategy, and sex demonstrated clear risk-stratified differences in predicted outcomes (Figure 4).

## Tricuspid regurgitation grade

The echocardiographic analysis at the 1-year follow-up was limited to the 87 patients with available follow-up echocardiography and, therefore, represents an imaging follow-up subset of the overall cohort. Consequently, these findings should be interpreted separately from the survival analyses, which included all 104 patients.

**Figure 5:**
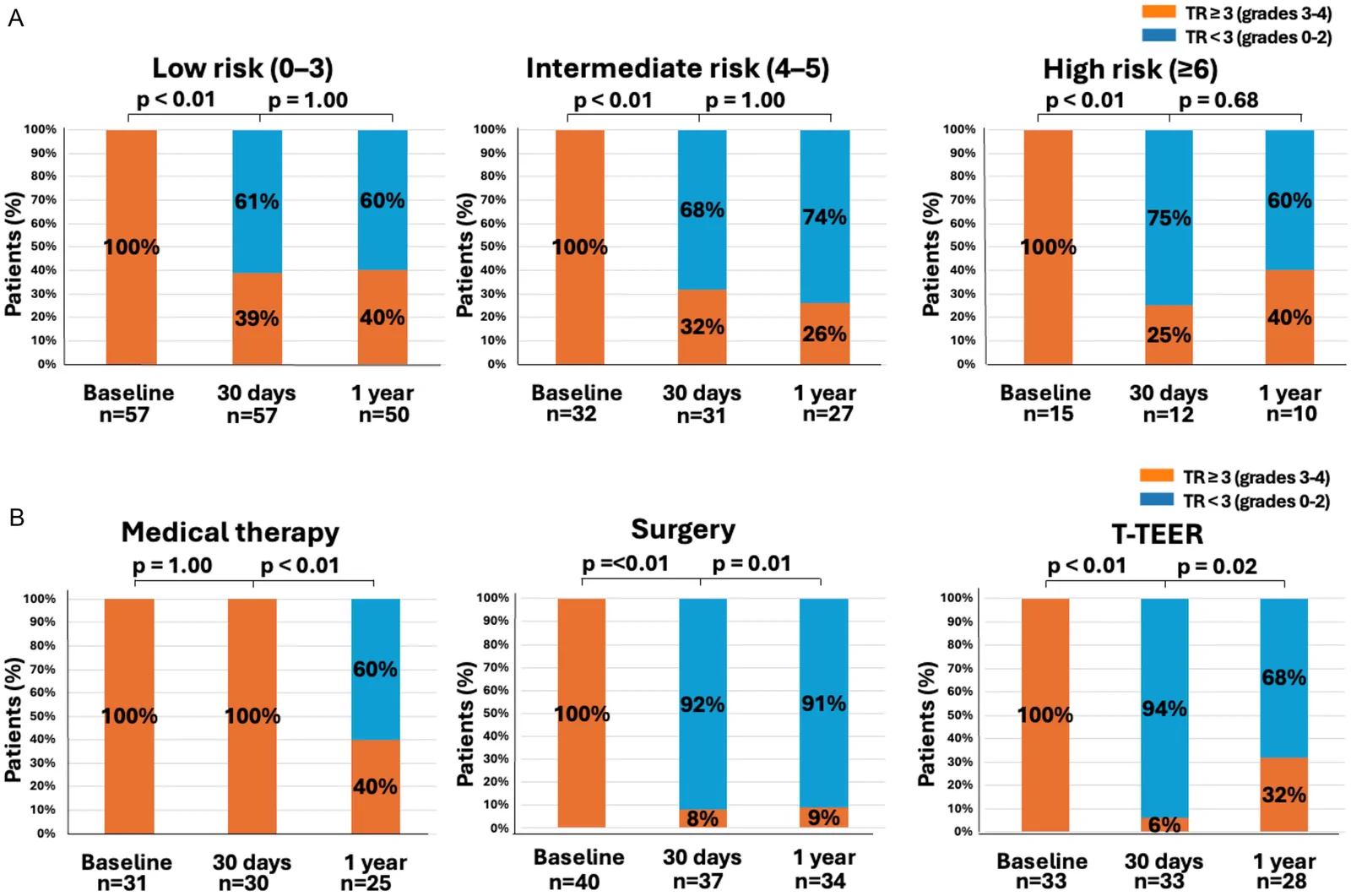
Longitudinal changes in tricuspid regurgitation severity at baseline, 30 days, and 1 year according to TRI-SCORE risk category and treatment strategy. *P* values were calculated using the paired Wilcoxon signed-rank test among patients with echocardiographic assessments available at both time points included in each comparison. These analyses were restricted to patients with available follow-up echocardiography and should be interpreted separately from the survival analyses performed in the entire study cohort. **(A)** Evolution of tricuspid regurgitation severity according to TRI-SCORE risk category. *P* values represent within-group comparisons between baseline and 30 days and between 30 days and 1 year. Sample sizes at each time point are shown below the bars. **(B)** Evolution of tricuspid regurgitation severity according to treatment strategy. *P* values represent within-group comparisons between baseline and 30 days and between 30 days and 1 year. Sample sizes at each time point are shown below the bars.

A marked and sustained reduction in TR to < moderate severity throughout the 1-year follow-up was observed in the low- and intermediate-risk TRI-SCORE categories (Figure 5A). At 1 year, 60% of patients in the medical therapy group had < moderate TR, compared with 68% and 91% in the T-TEER and surgical groups, respectively (Figure 5B). Descriptively, the greatest reduction in residual TR at 1 year was observed in the surgical group, followed by the T-TEER and medical therapy groups.

## Discussion

In this multicenter cohort of patients with isolated ≥ moderate tricuspid regurgitation (TR) managed with medical therapy, isolated tricuspid valve surgery, or T-TEER, the TRI-SCORE demonstrated consistent and independent prognostic value for both all-cause mortality and the composite endpoint of all-cause mortality or heart failure hospitalization. Higher TRI-SCORE values were associated with progressively worse outcomes irrespective of treatment strategy, supporting its role as a practical tool for baseline risk stratification across contemporary management pathways. These findings suggest that the TRI-SCORE primarily reflects the severity of underlying right-sided heart disease and systemic involvement rather than the effectiveness of any specific treatment strategy.

The TRI-SCORE was originally developed to predict in-hospital mortality after isolated tricuspid valve surgery and subsequently validated in patients undergoing redo tricuspid valve surgery, demonstrating excellent discrimination in surgical populations (14, 15). Our findings are consistent with those reported by Omran et al., who first demonstrated that the surgically derived TRI-SCORE retained prognostic value in patients undergoing transcatheter tricuspid interventions (16). Similarly, Gröger et al. showed that the TRI-SCORE outperformed EuroSCORE II and the Society of Thoracic Surgeons (STS) score in predicting mortality after T-TEER (17), while Adamo et al. confirmed its prognostic value in the TriValve registry (18). Together, these studies, culminating in the international TRIGISTRY registry, established the prognostic value of the TRI-SCORE across contemporary treatment strategies (16–20). Rather than introducing a new risk model, our study provides independent external validation in a separate multicenter cohort and extends these findings to a broader clinical population. Whereas the TRIGISTRY registry focused on patients with severe isolated functional tricuspid regurgitation (19), our cohort comprised patients with isolated ≥ moderate tricuspid regurgitation and a broader spectrum of disease etiologies, including functional, primary and CIED-related (organic), and mixed tricuspid regurgitation. This more heterogeneous population better reflects the diversity of patients encountered in routine clinical practice and further supports the applicability and generalizability of the TRI-SCORE across a wider clinical spectrum. In addition, our study characterized longitudinal changes in residual tricuspid regurgitation following medical therapy, isolated surgery, and T-TEER, complementing previous prognostic studies (19, 20) with descriptive information on treatment-related echocardiographic evolution during routine clinical follow-up. These observations are also consistent with the findings of the international TRIGISTRY registry, in which progressively worse survival was observed with increasing TRI-SCORE irrespective of treatment strategy (19). The investigators further demonstrated that the clinical benefit associated with successful intervention was greatest among patients with lower baseline risk, whereas outcomes remained poor in those with advanced disease (19). A subsequent analysis from the same collaborative registry further showed that the long-term benefit of isolated tricuspid valve surgery progressively declined with increasing TRI-SCORE, emphasizing that advanced disease stage substantially influences treatment outcomes regardless of intervention (20).

Our findings are consistent with these previous observations. Similar to prior reports, patients with high TRI-SCORE values experienced markedly worse outcomes regardless of treatment modality, supporting the concept that the TRI-SCORE principally reflects disease stage rather than treatment selection (19, 20). Furthermore, although outcome differences between the low- and intermediate-risk groups were relatively modest, patients classified as high risk had substantially higher rates of all-cause mortality and heart failure hospitalization. These findings suggest that the greatest clinical utility of the TRI-SCORE may lie in identifying patients who have already progressed to advanced right-sided heart failure and systemic organ involvement (19, 20).

Although EuroSCORE II also increased progressively across TRI-SCORE categories in our cohort, the two scores serve different clinical purposes. EuroSCORE II was developed to estimate perioperative mortality in patients undergoing cardiac surgery, whereas the TRI-SCORE was specifically designed to capture the clinical consequences of isolated tricuspid regurgitation by integrating markers of right-sided heart failure, renal dysfunction, hepatic congestion, and right ventricular dysfunction (14). These findings suggest that the TRI-SCORE provides disease-specific prognostic information that complements conventional surgical risk assessment, particularly in multidisciplinary Heart Team decision-making involving medical therapy, surgery, and transcatheter interventions.

Importantly, the present findings should not be interpreted as demonstrating the superiority of one treatment modality compared with other treatment strategies. Although adjusted model-based analyses suggested more favorable outcomes among surgically treated patients, treatment allocation was determined by multidisciplinary assessment rather than randomization and therefore reflected differences in age, comorbidities, anatomical suitability, right ventricular remodeling, operative risk, and overall clinical condition. Consequently, comparisons between treatment strategies remain subject to confounding by indication and should be considered exploratory rather than causal. Instead, these findings reinforce the importance of baseline disease stage as a major determinant of long-term prognosis irrespective of treatment strategy.

An additional descriptive finding of the present study was the difference in residual TR severity across treatment strategies. In our cohort, the greatest reduction in residual TR was descriptively observed among surgically treated patients, followed by T-TEER and conservative medical therapy. This observation should not be interpreted as evidence of superior treatment efficacy because of the observational, nonrandomized study design. Although 60% of medically managed patients demonstrated an improvement in TR severity to less than moderate at the 1-year follow-up, this finding should be interpreted cautiously. Medical therapy does not directly correct the underlying structural or functional mechanism responsible for TR. Rather, optimization of volume status with diuretic therapy, together with treatment of heart failure, rhythm control when appropriate, and contemporary guideline-directed medical therapy (8), including sodium-glucose cotransporter-2 inhibitors (SGLT2 inhibitors), may temporarily reduce right-sided filling pressures and tricuspid annular dilatation, thereby decreasing the echocardiographic severity of functional TR (8). However, because the underlying valvular, right ventricular, or right atrial pathology remains unchanged, these improvements are generally not expected to be durable in the absence of structural intervention (8).

In addition, because residual TR severity was evaluated only in patients with available follow-up echocardiography, the echocardiographic analysis represents a subset of the overall study cohort and should therefore be interpreted separately from the survival analyses, which were performed in the entire cohort. Selection bias related to the availability of follow-up imaging, together with the retrospective study design and inherent echocardiographic measurement variability, may also have contributed to the observed reduction in TR severity. The greatest reduction in residual TR was observed among surgically treated patients, followed by T-TEER, consistent with findings from contemporary surgical and transcatheter studies (9–13, 22, 23). Although the more sustained reduction in residual TR observed among surgically treated patients may represent one possible explanation for the more favorable adjusted model-based outcomes observed in this cohort, this observation should be considered hypothesis-generating rather than evidence of treatment superiority because of the non-randomized study design and the potential for residual confounding. Our findings should be interpreted in the context of the TRILUMINATE pivotal trial, which demonstrated significant improvements in tricuspid regurgitation severity, symptoms, quality of life, and functional status following T-TEER, without a corresponding reduction in mortality (10). Together with our findings, these results suggest that procedural success and reduction in residual TR alone may not fully overcome the adverse prognosis associated with advanced right-sided heart failure. Rather, long-term outcomes appear to be strongly influenced by baseline disease severity, underscoring the importance of appropriate patient selection and timely intervention (5, 8–10, 19). Overall, these observations highlight the complex interplay between baseline disease severity, treatment selection, procedural success, and subsequent clinical outcomes.

From a clinical perspective, these findings support incorporating the TRI-SCORE into baseline risk stratification to facilitate disease staging and inform multidisciplinary Heart Team decision-making. In addition, the descriptive longitudinal assessment of residual tricuspid regurgitation across medical therapy, isolated surgery, and T-TEER provides complementary insights into treatment-related echocardiographic evolution in routine clinical practice. Patients with lower TRI-SCORE values may represent an earlier stage of disease at which intervention may be associated with more durable reduction in tricuspid regurgitation and more favorable long-term outcomes. However, because this was an observational study, these findings should not be interpreted as evidence of comparative treatment efficacy. Rather, they underscore the importance of timely referral before irreversible right ventricular dysfunction and end-organ damage develop, consistent with contemporary guideline recommendations (5, 8). Conversely, patients with high TRI-SCORE values may represent a population with advanced cardiac and systemic disease in whom the potential benefits of intervention should be carefully balanced against procedural risk and the expected clinical benefit. This study was not designed to compare the efficacy of different treatment strategies.

## Limitations

This study has several limitations. First, its retrospective, observational, and nonrandomized design is inherently subject to selection bias, confounding by indication, and survivor bias, as treatment strategies were determined by multidisciplinary Heart Team judgment rather than randomization. Baseline for patients undergoing surgery or T-TEER was defined as the time of intervention to reflect the intended clinical application of the TRI-SCORE as a preprocedural risk assessment tool. Consequently, only patients who survived until intervention were included in these treatment groups, which may have introduced survivor (selection) bias. Therefore, treatment-related hazard ratios and comparisons between management strategies should be interpreted as exploratory associations rather than causal estimates of treatment effect. In addition, substantial baseline differences existed between treatment groups, including age, comorbidity burden, TR severity, EuroSCORE II, atrial fibrillation prevalence, and diuretic use, which may have influenced both treatment selection and clinical outcomes despite multivariable adjustment. Furthermore, changes in TR severity during follow-up may have been influenced by variations in loading conditions, optimization of medical therapy, and interobserver variability in echocardiographic assessment, particularly among medically managed patients, and should therefore be interpreted cautiously.

Second, the study was conducted in a moderate-sized multicenter cohort, and the relatively small sample size and limited number of outcome events reduced the statistical power, limiting the precision of the estimated hazard ratios and the robustness of subgroup and treatment-specific analyses. In addition, the limited number of patients within certain tricuspid regurgitation etiologic subgroups precluded meaningful etiology-specific analyses. To minimize the risk of overfitting, multivariable models were deliberately restricted to a small number of clinically relevant variables. Consequently, treatment comparisons should be considered hypothesis-generating rather than definitive. Furthermore, the sample size and number of outcome events precluded robust assessment of calibration and time-dependent discrimination. These limitations, together with the treatment-related findings, require confirmation in larger prospective multicenter cohorts.

Third, echocardiographic assessment was performed as part of routine clinical practice without a centralized core laboratory, introducing the potential for interobserver variability. In addition, clinical outcomes were collected retrospectively and were not adjudicated by an independent clinical events committee. Consequently, outcome definitions, particularly heart failure hospitalization, may be subject to variability inherent to retrospective data collection.

Fourth, a potential temporal bias should be acknowledged. Surgical patients were enrolled over a longer period beginning in 2010, whereas transcatheter therapies became available later during a period of rapid evolution in procedural techniques, device technology, operator experience, and contemporary medical therapy. These temporal differences may have influenced procedural success and long-term outcomes. In addition, follow-up duration varied among patients because of the retrospective study design, and patients without an event were censored at their last available clinical follow-up. Consequently, the number of patients at risk decreased over time, and estimates at later follow-up should be interpreted with appropriate caution.

Finally, although the TRI-SCORE demonstrated consistent prognostic value across the three management strategies evaluated in this study, the present analysis was not designed to determine the optimal treatment modality or establish causal treatment effects. Rather, our findings complement the existing evidence supporting the prognostic utility of the TRI-SCORE in a multicenter cohort that included patients with isolated ≥ moderate tricuspid regurgitation and a broader spectrum of disease etiologies. Accordingly, these findings should be interpreted as extending and complementing the existing evidence supporting the TRI-SCORE rather than establishing new evidence for treatment selection. Prospective multicenter studies with standardized imaging protocols, centralized event adjudication, and longer follow-up are warranted to further evaluate the performance of the TRI-SCORE across diverse patient populations and to clarify the prognostic significance of longitudinal changes in residual tricuspid regurgitation following different treatment strategies.

## Conclusion

In this multicenter retrospective cohort of patients with isolated ≥ moderate tricuspid regurgitation, the TRI-SCORE independently predicted all-cause mortality and the composite endpoint of all-cause mortality or heart failure hospitalization across patients managed with medical therapy, isolated tricuspid valve surgery, or T-TEER. These findings provide independent external validation of the TRI-SCORE in a contemporary multicenter real-world cohort encompassing a broader clinical and etiological spectrum of isolated tricuspid regurgitation, supporting its use for baseline risk stratification across contemporary management strategies. Because treatment allocation was observational and nonrandomized, comparisons between treatment strategies should be interpreted as exploratory rather than causal.

## Data Availability

The raw data supporting the conclusions of this article will be made available by the authors, without undue reservation.
