# Supplementary material for: TRI-SCORE based risk stratification across contemporary treatment strategies for isolated tricuspid regurgitation: a multicenter retrospective analysis
Source: Front Cardiovasc Med. 2026 Aug 26;13:1892142. doi: 10.3389/fcvm.2026.1892142 (PMC13553916; doi:10.3389/fcvm.2026.1892142)
Supplement: Supplementary file 1 [file Datasheet1.docx]

**Supplementary Materials**

**TRI-SCORE Based Risk Stratification Across Contemporary Treatment Strategies for Isolated Tricuspid Regurgitation: A Multicenter Retrospective Analysis**

Sara Gharehdaghi^1,2^, Liesbeth Rosseel^1^, Monika Beles^1^, Marc Vanderheyden^1^, Bernard Stockman^1^, Filip Casselman^1^, Raffaella Mistrulli^1,3^, Ivan Degrieck^1^, Karel Van Praet^1^, Jerrold Spapen^1^, Sofie Brouwers^1^, Frank Van Praet^1^, Martin Penicka^1^, Guy Van Camp^1^

1. AZORG Hospital, Cardiovascular Center, Aalst, Belgium

2. Semmelweis University, Doctoral School, Budapest, Hungary

3. Department of Clinical and Molecular Medicine, Sapienza University of Rome, Roma, Italy

**Corresponding Author:**

Guy Van Camp, MD, PhD

Cardiovascular Center Aalst,

AZORG Hospital,

Moorselbaan 164,

9300 Aalst,

Belgium

Email: [guy.van.camp@azorg.be](mailto:guy.van.camp@azorg.be)

**Table of Contents**

**Supplementary Table S1. TRI-SCORE risk factors and assigned points according to the original TRI-SCORE model described by Dreyfus et al. [14].**

**Supplementary Table S2. Distribution of TRI-SCORE components according to treatment strategy.**

**Supplementary Table S3. Baseline characteristics stratified by TRI-SCORE risk category.**

**Supplementary Table S4. Univariable and multivariable Cox proportional hazards models for all-cause mortality.**

**Supplementary Table S5. Univariable and multivariable Cox proportional hazards models for the composite endpoint of all-cause mortality and heart failure hospitalization.**

| **Risk factors for in-hospital mortality in patients undergoing isolated tricuspid valve surgery** | **Score** |
| --- | --- |
| **Age ≥ 70 years** | 1 |
| **NYHA functional class III–IV** | 1 |
| **Right-sided heart failure signs** | 2 |
| **Daily dose of furosemide ≥ 125 mg** | 2 |
| **Glomerular filtration rate < 30 mL/min** | 2 |
| **Elevated total bilirubin** | 2 |
| **LVEF < 60%** | 1 |
| **Moderate/severe RV dysfunction** | 1 |
| **Total possible score** | **12** |

**Supplementary Table S1.** TRI-SCORE risk factors and assigned points according to the original TRI-SCORE model described by Dreyfus et al. [14].

**Abbreviations:** NYHA, New York Heart Association; LVEF, left ventricular ejection fraction; RV, right ventricular.

| **Criterion** | **Medical (n/N, %)** | **Surgery (n/N, %)** | **T-TEER (n/N, %)** | ***P*-value** |
| --- | --- | --- | --- | --- |
| **Age ≥ 70 years** | 29 / 31 (93.5 %) | 26 / 40 (65.0 %) | 32 / 33 (97.0 %) | <0.01 |
| **NYHA class III–IV** | 6 / 31 (19.4 %) | 0 / 40 (0.0 %) | 9 / 33 (27.3 %) | <0.01 |
| **Right-sided heart-failure signs** | 2 / 31 (6.5 %) | 4 / 40 (10.0 %) | 9 / 33 (27.3 %) | 0.04 |
| **High-dose diuretics (≥ 125 mg furosemide eq.)** | 6 / 31 (19.4 %) | 13 / 40 (32.5 %) | 5 / 33 (15.2 %) | 0.2 |
| **eGFR < 30 mL/min/1.73 m²** | 6 / 31 (19.4 %) | 9 / 40 (22.5 %) | 3 / 33 (9.1 %) | 0.3 |
| **Elevated total bilirubin >1.2 mg/dL** | 4 / 31 (12.9 %) | 14 / 40 (35.0 %) | 1 / 33 (3.0 %) | <0.01 |
| **LVEF < 60 %** | 16 / 31 (51.6 %) | 20 / 40 (50.0%) | 4 / 33 (12.1 %) | <0.01 |
| **Moderate/severe RV dysfunction** | 11 / 31 (35.5 %) | 25 / 40 (62.5 %) | 14 / 33 (42.4 %) | 0.06 |

**Supplementary Table S2. Distribution of TRI-SCORE components according to treatment strategy.**

Values are presented as n (%). *P* values were derived using the χ² test to compare the three treatment groups.

Table S2 summarizes the distribution of the eight components of the TRI-SCORE across patients managed with surgery, transcatheter tricuspid edge-to-edge repair (T-TEER), or medical therapy. Older age, advanced New York Heart Association (NYHA) functional class, and signs of right-sided heart failure were more common among patients undergoing T-TEER or receiving medical therapy. In contrast, surgically treated patients had a lower prevalence of these high-risk features but a higher prevalence of reduced left ventricular systolic function and elevated bilirubin levels. Overall, the distribution of the TRI-SCORE components differed across treatment strategies, reflecting the heterogeneity in disease severity and clinical risk profiles among patients with isolated tricuspid regurgitation.

**Abbreviations:** T-TEER, transcatheter tricuspid edge-to-edge repair; NYHA, New York Heart Association; eGFR, estimated glomerular filtration rate; LVEF, left ventricular ejection fraction; RV, right ventricular.

| **Criterion** | **Total (N=104)** | **Low TRI-SCORE (N=57)** | **Intermediate TRI-SCORE (N=32)** | **High TRI-SCORE (N=15)** | ***P*-value** |
| --- | --- | --- | --- | --- | --- |
| **Age*****, years (median [IQR])** | 77.0 [69.8–83.0] | 76.0 [69.0–83.0] | 79.0 [73.3–83.0] | 80.0 [73.5–81.5] | 0.35 |
| **Male sex, n (%)** | 40 (38.5) | 22 (38.6) | 11 (34.4) | 7 (46.7) | 0.72 |
| **Length, cm** | 166 [161–170] | 165 [162–170] | 165 [160–169] | 168 [164–171] | 0.58 |
| **Weight, kg** | 72.0 [62.8–83.4] | 72.0 [64.0–82.0] | 72.5 [61.0–86.5] | 77.0 [63.0–83.8] | 0.96 |
| **Arterial hypertension, n (%)** | 48 (46.2) | 23 (40.4) | 20 (62.5) | 5 (33.3) | 0.07 |
| **Diabetes mellitus, n (%)** | 18 (17.3) | 8 (14.0) | 8 (25.0) | 2 (13.3) | 0.38 |
| **Atrial fibrillation, n (%)** | 83 (79.8) | 43 (75.4) | 26 (81.2) | 14 (93.3) | 0.3 |
| **Chronic kidney disease, n (%)** | 29 (27.9) | 10 (17.5) | 13 (40.6) | 6 (40.0) | 0.04 |
| **eGFR*****, mL/min/1.73 m²** | 48.5 [37.0–63.0] | 53.0 [44.0–74.0] | 41.0 [29.8–53.0] | 44.0 [30.0–50.0] | < 0.01 |
| **Coronary artery disease, n (%)** | 29 (27.9) | 9 (15.8) | 13 (40.6) | 7 (46.7) | < 0.01 |
| **Previous CABG, n (%)** | 16 (15.4) | 5 (8.8) | 6 (18.8) | 5 (33.3) | 0.05 |
| **Previous MV surgery, n (%)** | 31 (29.8) | 17 (29.8) | 6 (18.8) | 8 (53.3) | 0.05 |
| **ACE-inhibitor use, n (%)** | 29 (27.9) | 13 (22.8) | 12 (37.5) | 4 (26.7) | 0.33 |
| **ARB use, n (%)** | 16 (15.4) | 8 (14.0) | 6 (18.8) | 2 (13.3) | 0.82 |
| **Beta-blocker use, n (%)** | 77 (74.0) | 43 (75.4) | 24 (75.0) | 10 (66.7) | 0.78 |
| **MRA use, n (%)** | 67 (64.4) | 31 (54.4) | 25 (78.1) | 11 (73.3) | 0.06 |
| **Diuretic use, n (%)** | 75 (72.1) | 33 (57.9) | 28 (87.5) | 14 (93.3) | < 0.01 |
| **TRI-SCORE (baseline), median [IQR]** | 3.0 [1.0–4.25] | 2.0 [1.0–3.0] | 4.0 [4.0–5.0] | 6.0 [6.0–7.5] | <0.01 |
| **EuroSCORE II, median [IQR]** | 3.23 [1.69–5.95] | 1.92 [1.40–3.33] | 4.82 [3.24–8.59] | 9.76 [3.23–20.6] | <0.001 |
| **Functional TR, n (%)** | 86 (82.7) | 50 (87.7) | 25 (78.1) | 11 (73.3) | 0.30 |
| **Severe TR (grade 4), n (%)** | 58 (55.8) | 9 (15.8) | 13 (40.6) | 7 (46.7) | < 0.01 |

**Supplementary Table S3. Baseline patient characteristics stratified by TRI-SCORE risk category.**

Variables marked with an asterisk (*) are components of the TRI-SCORE. Consequently, statistically significant differences across TRI-SCORE risk categories are expected by construction and should not be interpreted as independent associations.

**Abbreviations:** ACEi, angiotensin-converting enzyme inhibitor; ARB, angiotensin II receptor blocker; CAD, coronary artery disease; CABG, coronary artery bypass grafting; CKD, chronic kidney disease; MRA, mineralocorticoid receptor antagonist; MV, mitral valve; TR, tricuspid regurgitation.

| **Predictor** | **Univariable model HR (95% CI)** | **Final Multivariable model HR (95% CI)** |
| --- | --- | --- |
| **TRI-SCORE (baseline)** | 1.27 (1.21–1.44) | 1.23 (1.08–1.40) |
| **Age (year)** | 1.06 (1.02–1.10) | 1.04 (1.00–1.08) |
| **Female sex** | 0.43 (0.21–0.84) | 0.45 (0.23–0.91) |
| **EuroSCORE II** | 1.07 (1.04–1.1) | — |
| **Treatment type** |  |  |
| **Medical** | Reference | — |
| **Surgery** | 0.48 (0.21–1.01) | — |
| **T-TEER** | 0.83 (0.36–1.93) | — |

**Supplementary Table S4. Univariable and multivariable Cox proportional hazards models for all-cause mortality.**

Clinically relevant candidate variables were first evaluated using univariable Cox proportional hazards regression analyses. Variables with P < 0.05 were considered eligible for inclusion in the multivariable model. Among these eligible candidate variables, age, sex, treatment strategy, and baseline TRI-SCORE were included in the initial multivariable model. EuroSCORE II was evaluated in the univariable analysis but was not entered into the multivariable model because of its conceptual overlap with TRI-SCORE, as both are composite risk scores that capture similar prognostic information. The remaining eligible covariates were sequentially removed using manual backward elimination with a predefined removal threshold of P > 0.05 to derive the final parsimonious model. TRI-SCORE was retained in all multivariable models irrespective of statistical significance because evaluation of its independent prognostic value was the primary objective of the study.

The table presents hazard ratios (HRs) with 95% confidence intervals (CIs) for predictors of all-cause mortality. Medical therapy served as the reference category for treatment strategy. In the final parsimonious multivariable model, treatment strategy was not retained, whereas higher baseline TRI-SCORE and increasing age were independently associated with a higher risk of all-cause mortality, and female sex was independently associated with a lower risk of all-cause mortality.

**Abbreviations:** HR, hazard ratio; CI, confidence interval; T-TEER, transcatheter tricuspid edge-to-edge repair.

**Footnote:** — indicates variables not retained in the final parsimonious multivariable model.

| **Predictor** | **Univariable model HR (95% CI)** | **Final Multivariable model HR (95% CI)** |
| --- | --- | --- |
| **TRI-SCORE (baseline)** | 1.30 (1.14–1.47) | 1.34 (1.17–1.52) |
| **Age (year)** | 1.05 (1.01–1.09) | — |
| **Female sex** | 0.43 (0.22–0. 82) | 0.48 (0.25–0.95) |
| **EuroSCORE II** | 1.06 (1.03–1.09) | — |
| **Treatment type** |  |  |
| **Medical** | Reference | Reference |
| **Surgery** | 0.42 (0.19–0.95) | 0.39 (0.17–0.89) |
| **T-TEER** | 0.77 (0.35–1.71) | 0.75 (0.34–1.66) |

**Supplementary Table S5. Univariable and multivariable Cox proportional hazards models for the composite endpoint of all-cause mortality or heart failure hospitalization.**

Clinically relevant candidate variables were first evaluated using univariable Cox proportional hazards regression analyses. Variables with P < 0.05 were considered eligible for inclusion in the multivariable model. Among these eligible candidate variables, age, sex, treatment strategy, and baseline TRI-SCORE were included in the initial multivariable model. EuroSCORE II was evaluated in the univariable Cox regression analysis but was not included in the multivariable model because of its conceptual overlap with TRI-SCORE, as both are composite risk scores that capture similar prognostic information. The remaining eligible covariates were sequentially removed using manual backward elimination with a predefined removal threshold of P > 0.05 to derive the final parsimonious model. TRI-SCORE was retained in all multivariable models irrespective of statistical significance because evaluation of its independent prognostic value was the primary objective of the study. For categorical variables retained in the final model, all corresponding levels are presented.

The table presents hazard ratios (HRs) with 95% confidence intervals (CIs) for predictors of the composite endpoint. Medical therapy served as the reference category for treatment strategy. In the final parsimonious multivariable model, higher baseline TRI-SCORE was independently associated with an increased risk of the composite endpoint, whereas female sex and surgical treatment were independently associated with a lower risk of the composite endpoint.

**Abbreviations:** HR, hazard ratio; CI, confidence interval; T-TEER, transcatheter tricuspid edge-to-edge repair.

**Footnote:** — indicates variables not retained in the final parsimonious multivariable model.
